# Supplementary material for: Reliability of Retinal Layer Annotation with a Novel, High-Resolution Optical Coherence Tomography Device: A Comparative Study
Source: Bioengineering (Basel). 2023 Mar 31;10(4):438. doi: 10.3390/bioengineering10040438 (PMC10136209; doi:10.3390/bioengineering10040438)
Supplement: Supplementary file 1 [file bioengineering-10-00438-s001.zip › bioengineering-2217034-supplementary.pdf]

Reliability of Retinal Layer Annotation with a Novel, High-Resolution Optical Coherence Tomography Device: A Comparative Study

Von der Emde et al. Bioengineering. 2023.

Supplemental table S1: Layer segmentation accuracy of intra-reader reliability with root mean squared error. Root mean squared error (RMSE) and standard deviation between duplicate grading by one reader. Values marked with an asterix are significantly smaller in the high-resolution data (α=0.05). Abbreviations: CHO: Choroid; BM: Bruch´s Membrane; RPE: Retinal pigment Epithelium; IZ: Interdigitation zone; EZ: Ellipsoid Zone; ELM: External limiting membrane; OPL: Outer plexiform layer; INL: Inner nuclear Layer; IPL: Inner plexiform layer; Ganglion cell layer; RNFL: retinal nerve fiber layer; ILM: Internal limiting membrane; High-Res OCT: investigational High-Res OCT; AMD: Age-related macular degeneration.

Modality	Group	Retinal layers											
		CHO	BM	RPE	IZ	EZ	ELM	OPL	INL	IPL	GCL	RNFL	ILM
High-Res OCT	AMD	23.3 ± 28.7	3.7 ± 1.6	4.9 ± 1.7	4.9 ± 1.8	2.8 ± 0.8	2.2 ± 0.5*	3.5 ± 0.9	3.1 ± 0.8	3.6 ± 0.8*	4.9 ± 2.5	3.2 ± 0.9	1.2 ± 1.9
	Control	20.8 ± 12.3	1.6 ± 1.9	2.6 ± 1.3*	3.2 ± 1.3	1.6 ± 0.4*	1.3 ± 0.4*	2.9 ± 0.6*	2.4 ± 0.9*	2.9 ± 0.7*	3.9 ± 1*	2.9 ± 0.8*	0.2 ± 0.8
Conventional_OCT	AMD	16.1 ± 13.1	4.9 ± 3.2	5.1 ± 1.5	5.7 ± 2	3.3 ± 1.4	3.3 ± 1.9	4.9 ± 3.8	3.6 ± 1.5	4.5 ± 1.3	5.3 ± 1.3	3.6 ± 0.9	0.3 ± 0.8
	Control	27.4 ± 19.3	1.7 ± 2.4	3.8 ± 1.2	3.4 ± 0.9	2.1 ± 0.5	2 ± 0.3	3.8 ± 1.1	3.4 ± 0.9	4 ± 0.7	6 ± 1.9	3.5 ± 0.7	0.6 ± 2.1

Reliability of Retinal Layer Annotation with a Novel, High-Resolution Optical Coherence Tomography Device: A Comparative Study

Von der Emde et al. Bioengineering. 2023.

Supplemental table S2: Layer segmentation accuracy of inter-reader reliability with the root mean squared error. Root mean squared error (RMSE) and standard deviation between two readers. Values marked with an asterix are significantly smaller in the high-resolution data (α=0.05). Abbreviations: CHO: Choroid; BM: Bruch´s Membrane; RPE: Retinal pigment Epithelium; IZ: Interdigitation zone; EZ: Ellipsoid Zone; ELM: External limiting membrane; OPL: Outer plexiform layer; INL: Inner nuclear Layer; IPL: Inner plexiform layer; Ganglion cell layer; RNFL: retinal nerve fiber layer; ILM: Internal limiting membrane; High-Res OCT: investigational High-Res OCT; AMD: Age-related macular degeneration.

Modality	Group	Retinal layers											
		CHO	BM	RPE	IZ	EZ	ELM	OPL	INL	IPL	GCL	RNFL	ILM
High-Res OCT	AMD	49.3 ± 36.3	3.8 ± 2.9	7.6 ± 4.3	7.3 ± 2.1*	5.1 ± 1.3	4 ± 1.4	6.2 ± 2.0*	4.4 ± 1.4*	4.7 ± 1.0*	6.9 ± 2.4	3.9 ± 1.3	1.5 ± 2.5
	Control	59.6 ± 53.5	0.9 ± 1.4	4 ± 1.4	4.6 ± 1.8*	4.8 ± 1.0	3.4 ± 2.7	6.5 ± 2.1	4.3 ± 2.0	4.9 ± 1.4*	6.4 ± 2.2*	3.4 ± 1.0	0.2 ± 0.8
Conventional_OCT	AMD	46.5 ± 23.4	5 ± 3.7	8.2 ± 4.5	7.9 ± 2.2	6.9 ± 2.6	4.2 ± 1.7	7.9 ± 4.1	5.0 ± 1.2	5.4 ± 1.4	10.6 ± 3	4.0 ± 1.0	0.1 ± 0.4
	Control	45.5 ± 21.5	1.2 ± 2.5	4.1 ± 1.5	3.8 ± 1.3	6.8 ± 1.6	3 ± 0.8	5.6 ± 2.3	4.4 ± 1.2	5.2 ± 1.5	11.1 ± 3.9	3.9 ± 1.4	0.4 ± 1.6
